# Studies on the breeding swarms of *Anopheles gambiae* complex in malaria control perspective

**DOI:** 10.1186/1475-2875-9-S2-O1

**Published:** 2010-10-20

**Authors:** Benoît S Assogba, Luc S Djogbenou, Roch K Dabiré, Abdoulaye Diabaté, Thierry Baldet

**Affiliations:** 1 IRSP/UAC, 01BP918Cotonou, Bénin; 2 CREC/IRD/UR016, 06BP2604Cotonou, Benin; 3 Centre Muraz/IRSS BP 390 Bobo-Dioulasso, Burkina-Faso

## Background

To reduce malaria transmission through vector control, alternative measures are necessary as transgenic mosquitoes are resistant to *Plasmodium *[1] and sterile insect technique (SIT) [2]. Both approaches require a fine understanding of the biology of reproduction of *Anopheles gambiae* complex. This study aims to characterize the swarm structure and several environmental parameters associated with distribution of breeding swams and sites in the south of Benin.

## Methods

After the survey in field, molecular analyses were done and productive breeding sites, breeding swarms and human habitations positions were integrated into a map using a geographic information system.

## Results

**Figure 1:**
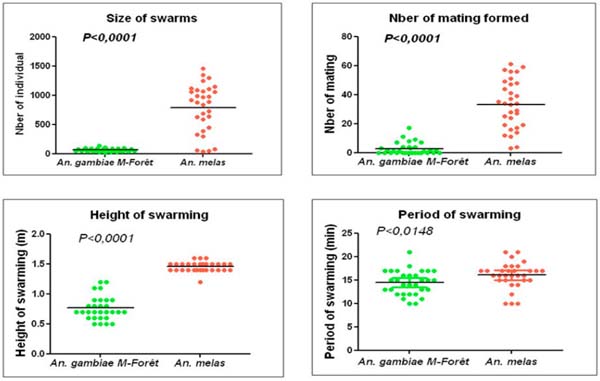
Comparison of swarm size, swarming period, height and number of mating couples observed between the swarms of *An. gambiae s.s.* M and *An. melas.*

**Figure 2:**
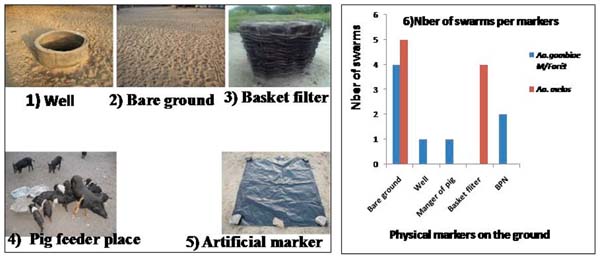
Physical markers of *An.gambiae* M and *An. melas* swarm sites.

**Figure 3:**
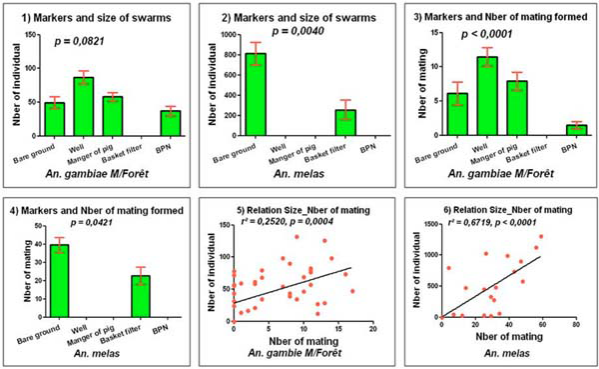
Relation between swarming markers and size and mating of swarms of *An. gambiae s.*

**Figure 4:**
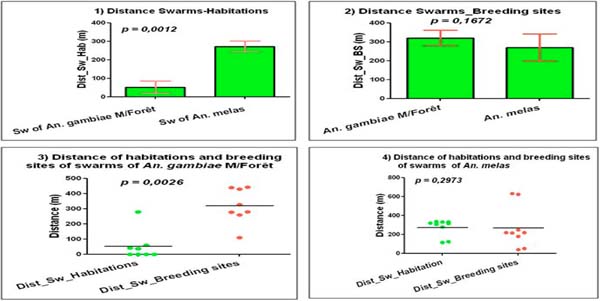
Relation between swarms, mosquito breeding sites and human house distribution in the study site.

**Figure 5:**
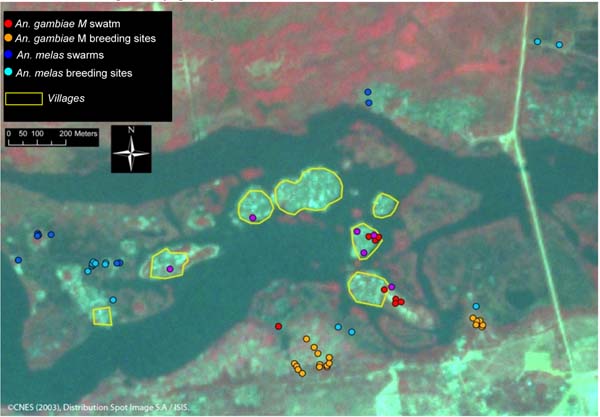
Distribution map of swarms and mosquito breeding sites of *An. gambiae s.s.* M and *An. melas* in the village of Djègbadji.

The molecular identification of 510 males collected from 17 swarms and 680 females from larvae of *An. gambiae s.l.* has allowed the description of breeding swarms and sites characteristic of two species: *An. gambiae* M and *An. melas* (Figures 1, 2,3) as well as their distribution (Figures 4, 5). These results indicate that the swarming and mating system involved mainly mosquitoes of the same species, suggesting the existence of specific factors related to the mating system of each species.

## Conclusion

Further studies on the swarming and mating system of *An. gambiae* s.l. need to be conducted to produce a predictive model of swarm distribution to aid malaria eradication strategies based on the use of generalized method of moments and SIT.
